# Address of Dr. P. G. C. Hunt

**Published:** 1864-08

**Authors:** 


### ADDRESS OF DR. P. G. C. HUNT,

**President of Ind. Dental Association.**

*Gentlemen of the Indiana State Dental Association:*

From the excited condition of the public mind and the necessary attention given to Military Preparations, it was thought necessary to postpone our Meetings for a time. At the suggestion of a number of members I have called the present Meeting, and hope the call will meet the approbation of all Members of the Association.

Gentlemen, let us remember "that it is good for brethren to dwell together in unity," that we may by social interchange of ideas be mutually benefited, and by so doing root up and cast aside those little heart-burnings and professional jealousies which tend so to mar the satisfaction and pleasure of professional usefulness.

We conceive that the relationship of dentistry and medicine is not, as a general thing, fully understoood or appreciated either by the profession or public. Ours is a speciality of the healing art. Dental Surgery is a legitimate branch of medicine. And we believe that a thorough knowledge of the one can not be attained without correctly understanding the other.

The public, some years ago, regarded dentistry simply me chanical, and that it required but little preparation to convert an ingenious mechanic into a competent dentist. But thanks to the regenerating influence of the egregious errors committed, this idea is in most communities obsolete. Community now require something more than a mere reputation as a mechanic.

Let us not be understood as depreciating the mechanic, on the contrary, we are of the opinion that the better the mechanic the more proficient the operator in general dental surgery, with an equal knowledge of the anatomical parts to be operated upon, and the remedial agencies to be applied.

There is not at this time the excuse for the student of dentistry not qualifying himself properly for his profession, that there existed a quarter of a century since. Then there existed but a meager amount of dental literature, and comparatively little knowledge to be had from private instructors. Now how different with our several dental colleges, their chairs filled with talent of the highest order. Talent that would do honor to any profession. And our numerous dental journals and periodicals so ably edited and so replete with useful knowledge. All these give the student of the present day great advantages, not enjoyed by those pioneers who labored so hard and industriously to lay the foundation of our ennobling profession. And a word to those of us, who have been a few years in practice.

With many dentists and physicians also, on leaving their preceptors, or having gone through their collegiate course (as the case may be) conclude they have studied sufficient for life. Further exercise in acquiring knowledge of their profession is entirely superfluous, they have learned it all. Unfortunately this is too frequently the case. I once heard a physician, who had enjoyed a very fair country practice for 15 years, remark that he kept no library, that he did not subscribe for, or receive, any of the periodicals or journals, because when he graduated he was pronounced qualified to practice medicine. Touching his forehead he remarked, I have it all here. The inference is plain. He was a clever companionable man, but by his colleagues considered almost without the pale of the medical profession proper,—and bordering on that of empiricism; entirely ignorant of the current medical literature of the day, dosing out calomel and jalap as profusely as he did fifteen years ago. What mattered it to him if common diseases assumed new types, or new diseases made their appearance—he had been dubbed Doctor of Medicine, and was he not fully equal to the task of curing or killing his patients? It is a lamentable fact that we have too many such practioners in our own profession. Men who, when they left their preceptors, were, according to their own views (and perhaps their teachers also), fully qualified to meet all emergencies that might arise in general practice. Starting in business for himself, mentally exclaming, I am through my studies now; hereafter my mind and all my energies must be bent on accumulating broad acres and stocks. I will not subscribe for this or that periodical; can't afford it, and besides it would be an useless expenditure of money—and I have it all in my head. And thus he goes on accumulating perhaps property, but not knowledge of his profession, soon firmly believing he can not spare the time to read or study, and so moves along in his professional career "neither turning to the right or left in all his actions, having the almighty dollar in view," until, perchance, he finds his number of patients growing small and disgustingly less by degrees. His patients have discovered that, although he was years ago, for that day, a competent physician—but now unmistakably behind his competitors; and all because he has not kept pace with his profession. That his great aim in life was the accumulation of money; the paltry sum he could receive for this or that operation, and not how best he could perform such operation, or how best benefit his fellow-men, how mitigate the most suffering.

We hold that the man who has no higher aim or ambition than the mere dollars and cents in our profession is not to be trusted—he is unworthy the calling. Let the watchword be, onward to professional excellence. Life is short; let us be doing something while the day is our own. Produce a thought, evolve a principle, adapt mechanism to perfect our practice in the treatment of disease—a wide, wide field is open to us.

All we know is enough of our profession only to see how much is wanting to perfect our theory and practice. Let each dentist make it his aim to furnish the world with new facts; better methods of treating disease, or loss of parts; more correct principles of diagnosis, and each contribute a quota to the general advance. Now I firmly believe that a profession such as ours, is only capable of an aggregate advance. That, in order to obtain a higher elevation, we must all stand on the same level, and go hand in hand, and if to-day a member of our Association, by study, experiment, reason, observation, or even seeming accident, becomes possessed of knowledge which enables him to accomplish better professional results than others; he himself is not prepared, and will not in all probability, make any further advance until the great mass of dental skill is brought up to his own level.

Therefore, up, up, to the highest step yet attained, and when all are there we will be ready for another advance. And let me assure you, *that* advance will be made. Discard prejudice, it is hurtful—it is a clog to improvement—it is the old man of the mountain, and yet it is so cherished by nearly every one of us as seriously to obstruct our improvement. Well can I call to mind times and places where professionally I have erred, through misconceived opinions formed without due enquiry and observation, many times on hearsay and ex-parte testimony, sometimes swayed by the authoritive diction of some of the sages of our profession who are, alas, erring mortals as well as me. Therefore discard, as far as possible, prejudice, and whatever you find useful to yourself communicate to your neighbor; do not feel as though he, being your rival for public patronage, should be excluded from your help. Nothing else seems so out of place, in a liberal profession, as a desire to keep your own supposed better methods of practice from a knowledge of your professional compeers. Nor yet do we conceive that is necessary always, as may be the practice of some, to recount the successful results of cases. But a true medium should be observed, ready in professional intercourse to impart information, but not obtrusive. And your errors, mistakes and failures, are perhaps of equal import to the aggregate of knowledge as your successes. Most men are chary of telling their failures, and yet we all make them—and they should be recorded, remembered, reported, and discussed.

Finally, gentlemen, we meet here to compare practice, to receive and impart useful knowledge, to spend a few days in discussing scientific topics, and extend to each other that fraternal feeling which should characterize those who labor for the same end. Let us cast aside every feeling, but that of love, love for each other, love for our profession, and love of truth—for "great is truth and mighty above all things," upon truth must all science stand.

